# High-accuracy individual identification using a “thin slice” of the functional connectome

**DOI:** 10.1162/netn_a_00068

**Published:** 2019-02-01

**Authors:** Lisa Byrge, Daniel P. Kennedy

**Affiliations:** Department of Psychological and Brain Sciences, Indiana University, Bloomington, IN, USA; Department of Psychological and Brain Sciences, Indiana University, Bloomington, IN, USA

**Keywords:** Functional connectivity MRI, Individual differences, Single-subject fMRI, Resting state, Within-subject reliability

## Abstract

Connectome fingerprinting—a method that uses many thousands of functional connections in aggregate to identify individuals—holds promise for individualized neuroimaging. A better characterization of the features underlying successful fingerprinting performance—how many and which functional connections are necessary and/or sufficient for high accuracy—will further inform our understanding of uniqueness in brain functioning. Thus, here we examine the limits of high-accuracy individual identification from functional connectomes. Using ∼3,300 scans from the Human Connectome Project in a split-half design and an independent replication sample, we find that a remarkably small “thin slice” of the connectome—as few as 40 out of 64,620 functional connections—was sufficient to uniquely identify individuals. Yet, we find that no specific connections or even specific networks were necessary for identification, as even small random samples of the connectome were sufficient. These results have important conceptual and practical implications for the manifestation and detection of uniqueness in the brain.

## Author Summary

Patterns of functional connectivity are so distinct between different people that they can be used to predict individual identity with high accuracy. Here, we show that a strikingly small fraction of the functional connectome is actually needed to predict individual identity (as few as 40 functional connections from 64,620). We further show that although certain functional connections may be most informative, even small fractions of the connectome selected at random can be used to identify individuals, and that no specific connections or even networks are actually necessary. The results indicate that uniquely identifying signatures of brain functioning are widely distributed throughout the brain and can be detected in a much more compact manner than previously appreciated.

## INTRODUCTION

Efforts toward accurately detecting, parsing, and understanding individual differences in functional imaging data have grown tremendously in recent years. One key advance toward these goals is the recent finding that individuals can be identified using fMRI data (Finn et al., 2015, 2017; Miranda-Dominguez et al., 2014, 2017; Vanderwal et al., 2017; Waller et al., 2017; Peña-Gómez et al., 2017; Horien et al., 2018; Amico & Goñi, 2018). Because functional connectomes, which quantify coordination between different regions spanning the entire brain, are consistently more similar within individuals than across individuals (e.g., Birn et al., 2013; Laumann et al., 2015; Noble et al., 2017a; Gratton et al., 2018), it is possible to predict with extremely high accuracy which individual contributed a particular fMRIscan simply on the basis of maximal similarity to functional connectomes from different scans.

Characterizing the specific features underlying the success of the fingerprinting algorithm—how many or how few, and which, functional connections are needed to differentiate individuals—will contribute to our understanding of the expression of neurobiological uniqueness. However, thus far there have been only a few studies of the connectome features underlying these successful identifications: we now know that fingerprinting accuracy is particularly high when using a subset of functional connections located within specific high-level functional networks (Finn et al., 2015; Peña-Gómez et al., 2017) or between specific regions of interest (Peña-Gómez et al., 2017), and that high identification accuracy can also be obtained when connectomes are reconstructed using a procedure that effectively compresses each connectome in a way that maximizes uniqueness (Amico & Goñi, 2018). But the lower limits are as yet unknown: we do not yet know how many functional connections are necessary for uniquely identifying individuals and how many are sufficient, and whether there are any specific functional connections or high-level functional networks that are necessary for high accuracy.

Thus, here we examine the limits of high-accuracy individual identification from functional connectomes. We find that a strikingly small number of functional connections (edges)—as few as 40 out of 64,620—is sufficient for high fingerprinting accuracy when those edges are selected on the basis of their individual distinctness in an independent partition of the data. However, none of those edges—and indeed no specific edges and no specific networks at all—are necessary for near-perfect identification accuracy. Indeed, random selections of hundreds of edges are also sufficient for high accuracy, suggesting that uniqueness in the functional connectome, although particularly concentrated in certain areas, is diffusely distributed. Just as identity can be predicted using a fraction of all possible individually diagnostic information in many other domains—from a partial fingerprint or DNA segment or from a brief glimpse at an occluded face—individuals can be identified using only a “thin slice” of the functional connectome.

## RESULTS

Here we examine 3,333 functional connectomes constructed from 835 subjects (∼4 scans each) from the Human Connectome project (Van Essen et al., 2013) in a split-half design. First, we seek to learn how small a subset (or “thin slice”) of the connectome can provide high identification accuracy by including only those functional connections (edges) most likely to distinguish individuals. To determine individually diagnostic value, we ranked edges according to their ratio of mean across-subject variability and mean within-subject variability by using an independent half of the dataset. Using the other half of the dataset, we then attempted to predict individual identity by using a subset of the connectome containing only those most individually distinct edges, using numerous different parcellations.

### Identity Can Be Predicted Using a Very Small “Thin Slice” of the Connectome

**Figure 1.**
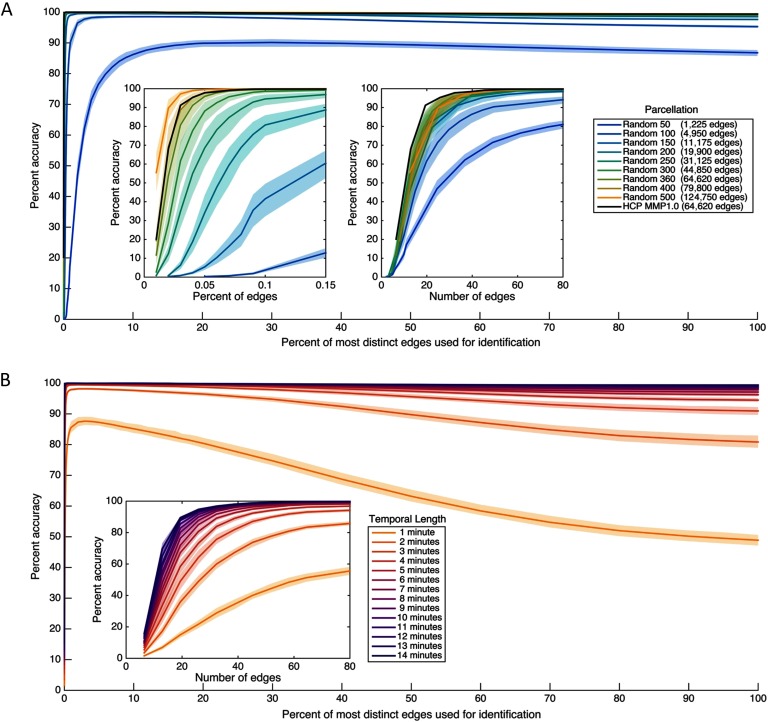
Individual identification accuracy as a function of how many of the most individually distinct edges in the functional connectome are used for identification. (A) Accuracy across different parcellations, expressed in terms of the percentage of the complete connectome used. For instance, x = 1 denotes using a subset of only the most distinct 1% of edges to identify individuals. Percentages are cumulative (e.g., the most distinct 2% includes the most distinct 1%) such that subset size changes as percent increases. The insets depict the same data rescaled to reveal very small *x*-axis values, with the left inset expressed in terms of the percentage of the complete connectome included (as in the main figure) and the right inset expressed in terms of the number of edges included in each subset. Note that the total number of edges varies across parcellations as indicated in the legend. (B) Accuracy across different contiguous temporal subsets of each scan, ranging from approximately 1 to approximately 13 min of data, using the HCP_MMP1.0 parcellation only. (The line for ∼14 min includes the entire scan rather than a temporal subset, and is a duplicate of the black line in Figure 1A presented for comparison.) Temporal subsets were taken from randomly selected initial TRs for each length examined (100 starting locations for temporal lengths up to 12 min; 18 starting locations, the maximum possible, for 13 min). Individual distinctness (i.e., individually diagnostic value) was independently computed using the corresponding temporal subset of the training partition (i.e., using the same number of TRs). The inset depicts the main figure rescaled to reveal very small *x*-axis values, and is expressed in terms of the number of edges rather than percentages. All plots depict 99% confidence intervals based on a bootstrap estimate of the mean across (a) five randomly selected parcellations of the same size or (b) five randomly selected initial TRs for the same temporal length.

The results, presented in Figure 1A, indicate that a remarkably small fraction of the full connectome under most parcellations examined is needed to identify individuals with near-perfect accuracy, with under 0.3% of all functional connections sufficient to provide effectively perfect accuracy (above 98%) for all but the three coarsest parcellation granularities. (See also Supporting Information Figure S1 for the same analysis conducted using a more stringent test of accuracy, in which the pattern of results is effectively the same despite slightly lower accuracy; Byrge & Kennedy, 2019.) Note also that neither head motion nor family structure is driving the pattern of results, which are the same when the analysis is conducted using different data quality thresholds (Supporting Information Figure S2, Byrge & Kennedy, 2019) and using a subset of only unrelated participants (Supporting Information Figure S3, Byrge & Kennedy, 2019). Figure 1A (inset, right) presents the same analysis rescaled as a function of the *number* of most distinct edges included in each connectome subset. Interestingly, despite the large range of connectome sizes examined (from 1,225 to 124,750 edges), accuracy appears to converge more across parcellations when similar *numbers* of edges rather than similar *fractions* of edges are used for prediction, with around 40–60 edges sufficient in most parcellations.

We replicate this same finding in a second independently collected dataset (coarsely parcellated; 114 regions of interests [ROIs]) composed of rest and video-watching scans from 48 individuals, using the same split-half design (half the subjects’ data for determining individually diagnostic value; half for prediction, with the ranking and prediction procedure conducted separately for rest and for video scans): 98% identification accuracy is achieved with a subset comprised of under 0.3% of the connectome for the rest scans (Supporting Information Figure S4, purple line, Byrge & Kennedy, 2019) and for the video scans (Supporting Information Figure S4, orange line, Byrge & Kennedy, 2019).

We also conducted this same primary thin-slice analysis while varying the temporal extent of the data included (i.e., the number of contiguous TRs), rather than parcellation size, by using the HCP_MMP1.0 parcellation only (Glasser et al., 2016) and using the HCP dataset here and for all subsequent analyses unless noted. These analyses were conducted separately within each temporal length (e.g., connectomes constructed using *X* TRs were used both for ranking and for prediction). The results, presented in Figure 1B, show a similar pattern: in most cases examined (for all but very short epochs), very high accuracy can be achieved using a very small fraction of the connectome (under 60/64,620 edges, or 0.09% of connectome, when at least 7 min of data are included, and under 194 edges, or 0.3%, for at least 3 min of data). Both Figure 1A and 1B also demonstrate that identification accuracy using connectome subsets can exceed accuracy for using the complete connectome (consistent with Finn et al., 2015), sometimes negligibly so, especially when near-ceiling performance is achieved, but considerably for coarse parcellations or short temporal extents.

**Figure 2.**
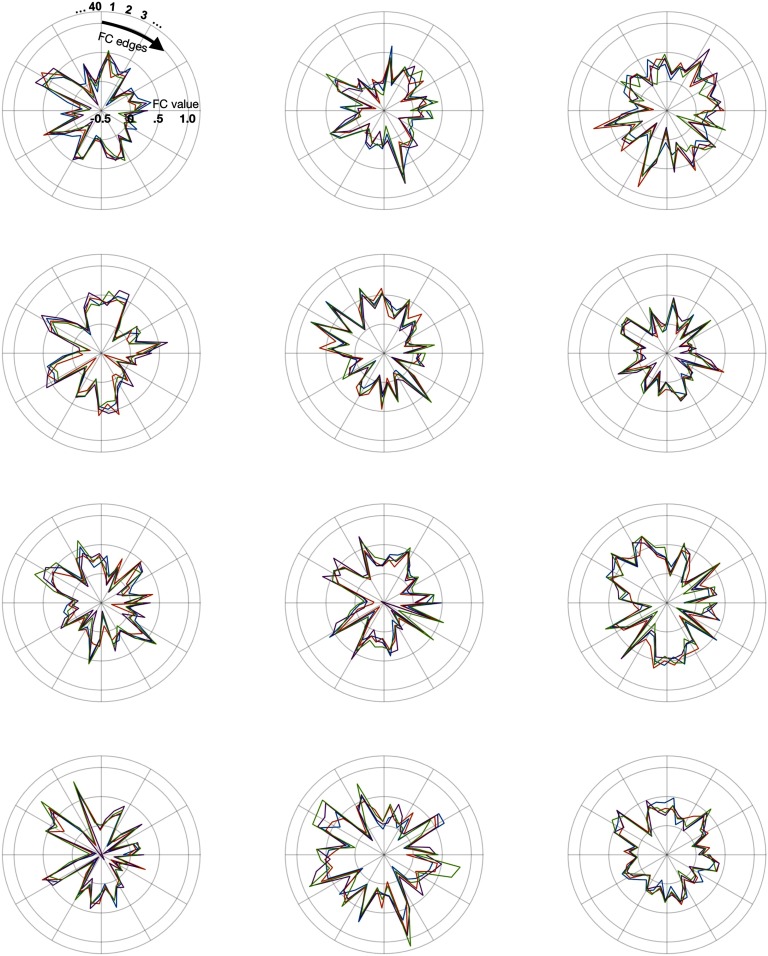
Examples of distinct patterns (i.e., fingerprints) of functional connectivity within individual subjects. Each plot presents data from one randomly selected subject, with data from their four different scans plotted in different colors. All plots depict functional connectivity values (Fisher *z*-transformed correlations) for the 40 edges with highest individual distinctness. To allow for visualization of negative correlation values, the radius extends from −0.5 at center to a maximum of 1.2; grid lines indicate 0, 0.5, and 1. See also Supporting Information Figure S5 (Byrge & Kennedy, 2019) for data from many other randomly selected subjects.

Overall, very high fingerprinting accuracy can be achieved with a tiny subset of the most diagnostic connectome edges. The structure driving these successful identifications (i.e., “fingerprints”) can be observed at a glance. Figure 2 presents the values for the most distinct 40 edges (from the HCP_MMP1.0 parcellation) for all scans from several example subjects (for even more subjects, see also Supporting Information Figure S5, Byrge & Kennedy, 2019). It is apparent that functional connectivity patterns across these edges are consistent within individuals and distinct from other individuals (see also Supporting Information Figure S6, Byrge & Kennedy, 2019, which separately examines within-individual similarity [or self-similarity] and across-individual similarity [or other-similarity] following Amico & Goñi, 2018; the gap between within and across- individual similarity is apparent and especially pronounced for high-ranked edges).

### Individually Distinct Information Is Widespread Throughout the Cortex and Concentrated in Fronto-Parietal Regions

**Figure 3.**
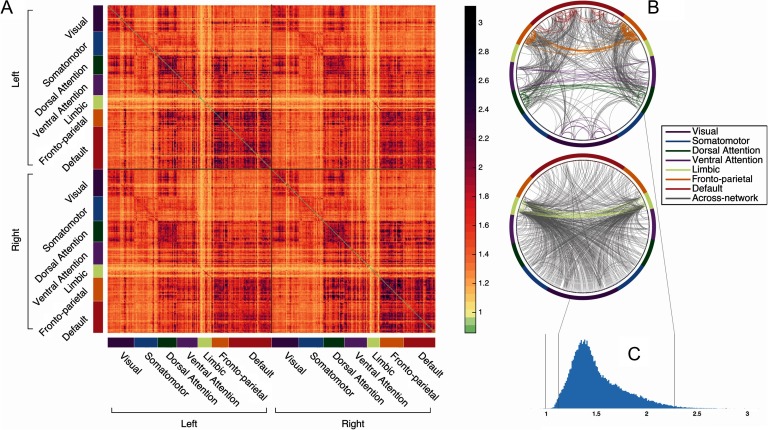
Individually diagnostic values for the HCP_MMP1.0 (Glasser et al., 2016) parcellation established using an independent partition of the dataset. Individually diagnostic value is computed here as the ratio of mean variability across subjects and mean variability within subjects, such that values above 1 indicate some individually distinct information (e.g., greater variability across than within subjects). (A) Individually diagnostic values for each edge, ordered to indicate large-scale functional membership (Yeo et al., 2011). As ratios, these values are unitless and indicate the magnitude with which across-subject variability exceeds within-subject variability (or vice versa); the high values indicate edges that are considerably more variable across subjects than within them. (B) Circle plots visualizing the 1% of edges with the highest (top) and the lowest (bottom) individually diagnostic value. Nodes and within-network edges are in color; across-network edges are in gray. (C) Histogram of all diagnostic values. Gray vertical lines indicate, from left to right, 1, the bottom 1%, and the top 1%. Note that nearly all edges exceed 1, indicating some individually distinct information. See also Supporting Information Figure S9 (Byrge & Kennedy, 2019) for similar information for the replication dataset.

Figure 3A depicts the individually diagnostic values computed to capture the distinctness of each functional connection (edge) in the HCP_MMP1.0 parcellation, calculated as the ratio of mean across-subject variability and mean within-subject variability in an independent partition of the dataset. (The data used to create this plot has been made available at brainlab.psych.indiana.edu/resources/hcpmmp10_diagnostic_values.mat). Figure 3B depicts the most distinct 1% of edges and least distinct 1% of edges in this parcellation. Consistent with other reports (Miranda-Dominguez et al., 2014; Finn et al., 2015; Laumann et al., 2015; Airan et al., 2016; Peña-Gómez et al., 2017; Gratton et al., 2018), edges that fall within fronto-parietal and default networks are particularly individually distinct. The most distinct edges tend to be intrahemispheric (Figure 3B) and within-network (purple edges in Figure 3A). Less-distinct edges appear concentrated in the limbic regions, which are located in areas with high artifact susceptibility near sinuses and subcortical structures and have a lower signal-to-noise ratio (Yeo et al., 2011). Supporting Information Figure S7 (Byrge & Kennedy, 2019) depicts the most and least distinct edges across several random parcellations; as is clear, these edges largely converge across parcellations, with the same networks consistently implicated as more informative or as less informative. Supporting Information Figure S8 (Byrge & Kennedy, 2019) presents the top-ranked edges for the primary dataset and for the replication rest dataset together for visual comparison, as well as the mean individually diagnostic values within and between each pair of high-level functional networks; although individual edges are not compared directly across these datasets because of data format differences (surface-based vs. volumetric), similarities are apparent and mean individually diagnostic values for high-level functional networks are highly correlated across datasets (*r* = 0.89, *p* << 0.001). See also Supporting Information Figure S9 (Byrge & Kennedy, 2019) for a comparison of the individually diagnostic values computed in the replication rest and replication movie dataset using the same selection of training partition subjects: rankings were generally similar (*r* = 0.74, *p* << 0.001) despite some modulation by task context (cf. visual and somatomotor network among top-ranked edges in movie but not rest data).

Finally, the distribution of individual diagnostic values (Figure 3C) reveals that the vast majority of edges—throughout the cortex—exceed 1 (median = 1.45 with 99.98% > 1 in the primary dataset; median = 1.57 with 99.97% > 1 in the replication rest dataset; median 1.63 with 99.8% > 1 in the replication video dataset), indicating some individual distinctness (e.g., more across-subject variability than within-subject variability, on average). This suggests that many connections (and not only the maximally ranked edges) might contribute to the identification of individuals.

### Identity Can Be Predicted Without Highly Distinct Functional Connections

**Figure 4.**
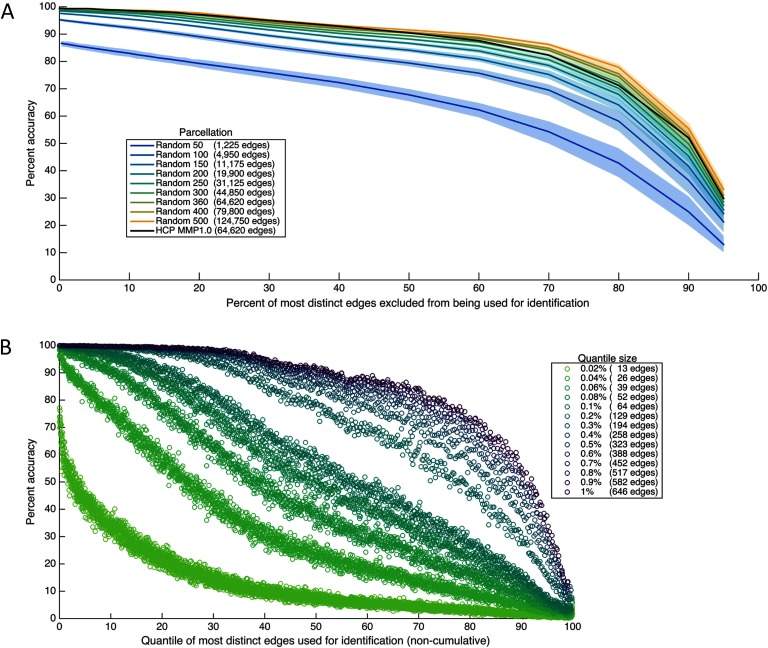
Identification accuracy when the most distinct connectome edges are not used for identification. (A) Accuracy as a function of how many of the most distinct edges in the functional connectome are excluded in each subset, across different parcellations, expressed in terms of the percentage of the complete connectome excluded in each subset. For instance, x = 1 denotes using a subset that excludes the most distinct 1% of edges to identify individuals (and instead includes the remaining, least distinct, 99% of edges). Percentages are cumulative, and subset size changes as percent increases. Confidence intervals of 99% based on a bootstrap estimate of the mean across five random parcellations of the same resolution are depicted for each random parcellation resolution. (B) Accuracy as a function of the quantile of most distinct edges used for identification, for different quantile sizes, using the HCP_MMP1.0 parcellation only. Quantiles are not cumulative (e.g., neighboring quantiles contain different edges) and are plotted on a scale of 0–100 for comparability even though most are smaller than percentiles.

To examine this possibility, we attempted to predict individual identity using the reverse of the first analysis: connectome subsets that *exclude* the most diagnostic edges. So, for instance, instead of predicting individual identity by using a subset of the most distinct 1% of edges (as in Figure 1A), here we predict individual identity by using a subset of all functional connections *except* the most distinct 1% (in other words, using the least distinct 99% of edges; see also Vanderwal et al., 2017, for a similar approach). The results, depicted in Figure 4A, indicate that although accuracy decreases as the most distinct edges are eliminated (as would be expected), the edges with the highest distinctness are not required for high identification accuracy. For the most fine-grained parcellations, successful fingerprinting can still be achieved even when the most distinct 15% of the connectome is omitted, using the least distinct 85% of the connectome to identify individuals.

In the previous analyses, the size of the connectome subset used for prediction has covaried with the distinctness of the edges included in that subset. To decouple these two dimensions, next, using the HCP_MMP1.0 parcellation only for this and subsequent analyses, we attempted to predict individual identity by using connectome subsets composed of quantiles of ranked edges, across several quantile sizes. So, more concretely, for percentiles (the coarsest quantile size examined), we first predicted identity using the 646 most distinct edges (the first 1%), and then using the 646 next most distinct edges (the second 1%), and so on. In this way the individually diagnostic value of edges within a subset is varied but the size of the subset remains fixed. The results, presented in Figure 4B, indicate again that the most distinct edges are not required for high identification accuracy, and furthermore that large connectome subsets are not required nor are any specific edges. Individual identity can be predicted with above 98% accuracy by using many different subsets composed of ∼400–650 functional connections (0.7%–1% of the connectome) even without using the most distinct 25% of the connectome.

### Identity Can Be Predicted Using Randomly-Selected “Thin Slices”

**Figure 5.**
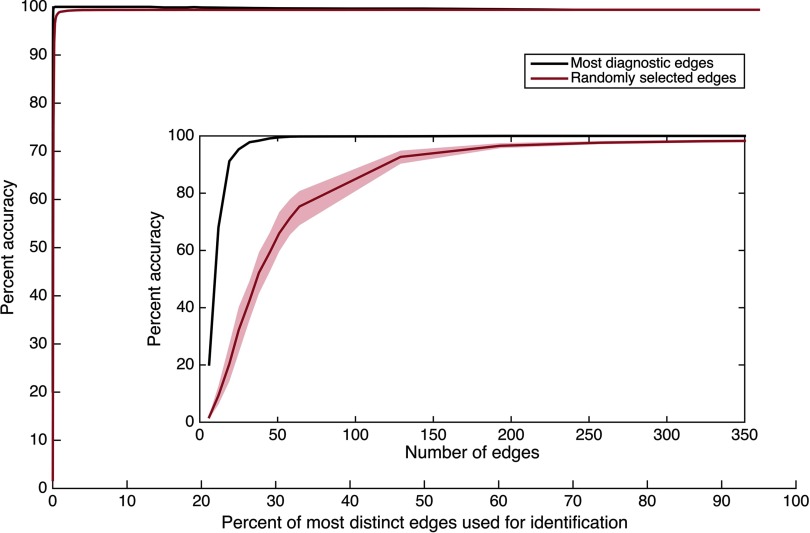
Identification accuracy as a function of how many randomly selected edges are included in each subset, using the HCP_MMP1.0 parcellation only. Percentages are cumulative (e.g., 2% contains twice as many edges as 1%). Confidence intervals of 99% based on a bootstrap estimate of the mean across five (out of 100) random edge selections for each percentage examined are displayed. The inset depicts the main figure rescaled to reveal very small *x*-axis values. See also Supporting Information Figure S6 (Byrge & Kennedy, 2019), which presents mean within-individual and across-individual similarity for these random connectome subsets.

These results indicate that no specific functional connection is required for high identification accuracy. The next question that arises is whether *any* sufficiently large subset of the functional connectome—with functional connections selected at random—might also provide high identification accuracy. The previous results hint at this possibility: virtually any random selection of edges from a distribution like Figure 3C will contain edges with some individual distinctness, and a sufficiently large selection will contain some of the more highly distinct edges that especially facilitate high accuracy (see Figure 1A). We thus attempt to identify individuals by using randomly selected subsets of the same sizes examined in Figure 1A, with 100 random selections per subset size. The results are presented in Figure 5 (along with the corresponding original results from Figure 1A, using the most distinct edges, re-presented for comparison). They indicate that although random edge selection underperforms ranked edge selection for very small subset sizes, the two selection methods quickly converge, and a random subset composed of 0.5% of the connectome (323 edges) achieves individual identification accuracy exceeding 98% on average.

### No Specific Functional Network, Functional Connection, or Set of Functional Connections Is Required for Identity Prediction

**Figure 6.**
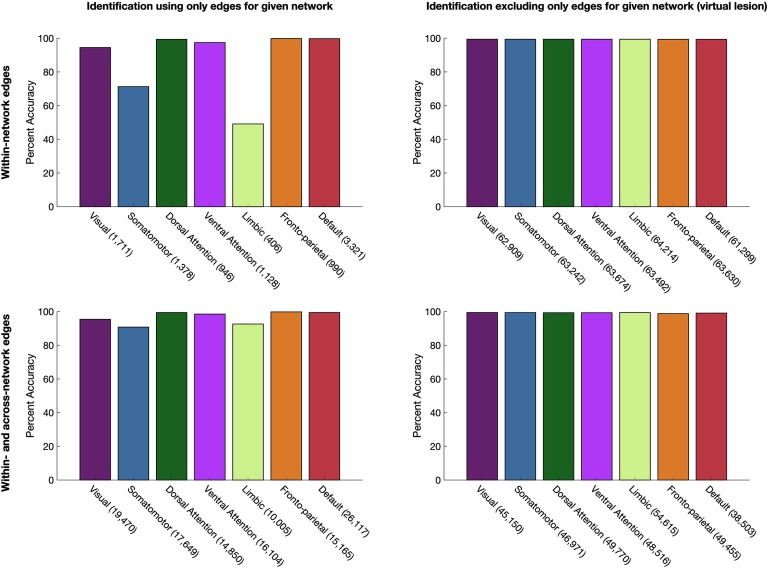
Identification accuracy using network-level connectome subsets. The number of edges included in each subset is listed in parentheses. Left column: identification using subsets composed of (top row) only within-network edges and (bottom row) only within- and across-network edges for each network. Right column: virtual lesion approach, with identification using subsets comprised of (top row) all connectome edges except within-network edges, and (bottom row) all connectome edges except within- and across-network edges for each network.

That randomly-selected subsets of edges considerably smaller than large-scale functional networks can identify individuals raises the possibility that perhaps no specific functional network is required for identifying individuals. We thus examined individual identification accuracy by using connectome subsets defined at the network level (using a data-driven parcellation of the cortex into 7 functional networks; Yeo et al., 2011). For each network, we examined identification accuracy by using subsets composed of only the edges associated with that network, and then, taking a virtual lesion approach, using subsets composed of the entire connectome *except* the edges associated with that network. In both cases, we repeated this analysis by using within-network edges exclusively, as well as within- and across-network edges for the given network. The results, presented in Figure 6, replicate previous findings (Finn et al., 2015; Peña-Gómez et al., 2017) of a gradient of identification accuracy when each network is used for identification in isolation (Figure 6, top), but provide a complementary perspective on those findings by showing that although several networks are sufficient for identification, no one network is *necessary* (Figure 6, bottom); even the fronto-parietal network can be explicitly excluded with high accuracy maintained. Altogether, these findings indicate that while signatures of individual variability may be particularly concentrated in some networks, regions, and edges, signatures of individual uniqueness can be detected throughout the cortex.

## DISCUSSION

Here we find that in this large and high-resolution fMRI dataset, individual identity can be predicted using a surprisingly tiny fraction—a “thin slice”—of the full functional connectome. High accuracy with the smallest possible connectome subset can be obtained using specifically those edges that were determined to be highly individually distinct in an independent partition of the data, and those highly distinct edges were largely located within fronto-parietal regions consistent with prior work (Miranda-Dominguez et al., 2014; Finn et al., 2015; Airan et al., 2016; Peña-Gómez et al., 2017; Gratton et al., 2018; Amico & Goñi, 2018). However, nearly all edges in the connectome contain some individually diagnostic information, and no specific edges or specific functional networks are necessary for predicting identity in this dataset. Effectively perfect accuracy can be obtained without the most distinct 10–15% of the connectome (when the remainder of the connectome is used for prediction) and even without the most distinct 25% of the connectome (when a small subset of moderately diagnostic edges is used for prediction). Perhaps most strikingly, a randomly selected subset smaller than 1% of the functional connectome can also provide effectively perfect identification. Individuals can thus be identified by not just one but many “thin slices” of the connectome.

Together these findings suggest that although individual variability in brain function may be particularly *concentrated* in fronto-parietal regions in accord with previous literature (Mueller et al., 2013; Miranda-Dominguez et al., 2014; Finn et al., 2015; Airan et al., 2016; Peña-Gómez et al., 2017; Gratton et al., 2018; Amico & Goñi, 2018), uniqueness in functional coupling patterns is far more diffusely distributed throughout the brain than previously appreciated. Less highlighted findings in earlier work hint at this possibility: identification accuracy using networks outside fronto-parietal regions (Finn et al., 2015), using random selections of ROIs (Peña-Gómez et al., 2017), and using the least informative edges in the connectome (Vanderwal et al., 2017), while below ceiling accuracy, far exceeded what would be expected by chance. Thus, just as time-locked task-based fMRI responses are revealed as far less localized and more diffusely distributed than previously appreciated when sampled in a different way (Gonzalez-Castillo et al., 2012), detecting individual uniqueness in fMRI data appears to be not exclusively a question of sampling from *specific* locations but also a question of sampling from *enough* functional interactions. In other words, we need to cast a net that can capture diffusely distributed individually diagnostic information, rather than a net that must land on specific locations.

Because we show here that individually diagnostic information from one independent group of subjects (from the training partition) contributes to identification accuracy in a different group of subjects (from the test partition), this implies that relative distinctness of connectome edges is similar across different groups of individuals, which is itself noteworthy. We also confirmed this post hoc and found highly correlated individually diagnostic values across training and test sets across the entire cortex (*r* = 0.972, *p* << 0.001) and within each individual network (mean *r* = 0.942 [*SD* = 0.031], all *p* << 0.001). This did not necessarily have to be the case: an alternative model for why connectome fingerprinting works is that if enough edges are sampled (either from the whole connectome or from large-scale distributed networks, like the fronto-parietal network), then enough individually distinctive patterns are obtained that can be used *in aggregate* to differentiate individuals. For example, edges 1–40 might be highly predictive of individual A, whereas edges 41–80 are highly predictive of individual B and edges 81–120 are predictive of individual C. And so, by sampling from edges 1–120 and beyond, we obtain enough information to differentiate all these individuals from one another. However, our results indicate that although such broad sampling is certainly *sufficient* to identify individuals (Figure 5), it is not *necessary*. Rather, although we find that individually distinctive information is widely distributed throughout the brain (see Figure 3A and 3C), the specific edges with the most or the least individually distinct edges are consistent *across* independent groups of subjects. Furthermore, although edges from the primary and replication datasets were not directly compared because of different data formats (surface-based vs. volumetric), a visual comparison of the top-ranked edges in the different datasets (Supporting Information Figure S8, Byrge & Kennedy, 2019) and of the mean ranking of within- and across-network edges (Supporting Information Figure S9, Byrge & Kennedy, 2019) are suggestive of considerable similarities in the relative distinctness of edges across the two datasets. Altogether these findings indicate that there may be a consistent, reproducible gradient of uniqueness across the connectome, such that some edges can play an outsized role in individual identification while others play a more minimal (and even detrimental; see below) role. We have made available the individually diagnostic values of edges using the HCP_MMP1.0 (Glasser) parcellation at brainlab.psych.indiana.edu/resources/hcpmmp10_diagnostic_values.mat.

Not only was the individually diagnostic information for different edges consistent between two groups of subjects, it was consistent within the training partition across 900 random parcellations of nine different granularities. As Supporting Information Figure S7 (Byrge & Kennedy, 2019) demonstrates, we observed a high degree of convergence across parcellations in the networks implicated in the most and least distinct edges. We also observed that same-sized connectome subsets obtain similar accuracy across different parcellations of the same granularity (cf. narrow confidence intervals in Figures 1A and 4A; also cf. black line for HCP_MMP1.0 parcellation and yellow-green line for random parcellations of the same size). Together these results indicate that precise boundaries between regions are not driving the pattern of results; in other words, individual distinctness of connectome edges arises broadly not as a result of how the data are aggregated but rather as a property of the underlying signals. (We note, however, that only group-level parcellations were employed in this study and that individually defined parcellations as described by Bijsterbosch et al. [2018] may be expected to further enhance accuracy, potentially with even smaller connectome subsets.)

Across training and test partitions, across random parcellations, and within the replication dataset, neuroanatomical locations of the nodes involved in the more distinct and in the less-distinct edges accord with the existing literature: more distinct edges involve locations with higher interindividual variability (Mueller et al., 2013; Laumann et al., 2015; Airan et al., 2016; Bijsterbosch et al., 2018; Gratton et al., 2018); less-distinct edges originate near sinuses and brainstem with their susceptibility to fMRI signal dropout and other artifacts (Yeo et al., 2011; Peña-Gómez et al., 2017). That the least informative edges may consistently contain increased fMRI noise is likely to explain the decrement in accuracy we observe when identifying individuals by using the complete connectome (see also Finn et al., 2015, who found that accuracy using combined fronto-parietal networks exceeds that of the whole connectome). Although this accuracy deficit is negligible for higher granularity parcellations and longer scans, it may have real practical consequences in applications where scan duration is limited or parcellations must be coarse (see also Airan et al., 2016; Waller et al., 2017; Horien et al., 2018).

Amico and Goñi (2018) have pointed out the importance of going beyond accuracy metrics and understanding the underlying similarity among connectomes in fingerprinting analyses—in principle, high identification accuracy could be obtained in datasets where scans are generally highly dissimilar and within-individual scans are also dissimilar, just slightly less so, potentially changing the interpretation of the results. Figure 2 (see also Supporting Information Figure S5, Byrge & Kennedy, 2019) demonstrates that such a scenario is unlikely in this dataset, given the visually evident similarity among connectome subsets from an individual and dissimilarity across individuals; Supporting Information Figure S6 (Byrge & Kennedy, 2019) shows that not only is within-individual similarity higher than across-individual similarity, within-individual similarity is itself very high. These similarity measures also speak to an apparent tension between our findings that individuals can be identified using mere minutes of data (Figure 1B) and other work showing that hours of data is necessary for a stable estimate of functional connectivity within an individual (Laumann et al., 2015): for the connectome subset sizes achieving high accuracy, the gap between within-individual similarity and across-individual similarity is substantial. Even though within-individual similarity would be expected to continue to increase given longer scan times, it would still be expected to be high after several minutes of data (cf. Figure 4 in Laumann et al., 2015), and sufficiently higher than across-individual similarity to permit identification. We note that it could also be the case that the within-individual similarity for the maximally distinct connectome subsets analyzed here might stabilize more quickly (i.e., requiring fewer hours of data), because the influence of less-distinct noisy edges would be eliminated.

That small connectome subsets selected at random can accurately identify individuals is likely to arise from a combination of two factors: sampling (random selections of 1% of all edges are likely to include some edges within the top tier of individually diagnostic value) and more widespread individual distinctness than previously appreciated. For instance, identity prediction using a small subset of connectome edges in the 70th percentile of individually diagnostic values can achieve accuracy exceeding 80% (Figure 4B)—dramatically higher than expected by chance—indicating considerable individually diagnostic information is present even in those relatively less distinct edges. An important question for future studies is whether it might be possible to better harness the individually diagnostic information present in edges with moderate-to-low distinctness. Perhaps with some modifications to the fingerprinting algorithm (potentially combining information across scans or changes to scan acquisition parameters), ceiling accuracy might be achievable using only those relatively less distinct edges as well. Alternatively—just as identification from actual fingerprints (from fingers) preferentially uses the most informative points or features—weighting connectome edges according to their (independently determined) distinctness prior to prediction might improve identification accuracy. Indeed, preliminary results with no attempt at optimization indicate that linearly weighting connectome edges can improve accuracy by around 5% for the coarsest parcellation examined (Supporting Information Figure S10, Byrge & Kennedy, 2019). Such an improvement may be particularly important for identification in extremely large datasets, for which accuracy might be expected to fall below ceiling level.

Whether these results have implications for biomarker development and other approaches for linking individual difference measures with their neural correlates is thus far an open question. These results do provide a reason for optimism—signals at the level of individual edges can be highly and precisely measured within approximately 15 min of scanning (an important feature for potential biomarkers)—and combining dozens of such edges are uniquely identifying. Thus, if there are signals that differentiate two groups reliably (e.g., clinical groups), or continuously track well-measured behavioral or cognitive traits, we should be able to detect them. However, it is also likely to be the case that identifying individuals from connectomes may be a less difficult task than identifying behavioral or clinical links. For example, individual neuroanatomy is more consistent across scans for the same individual compared with different individuals. Similarly, the identity of an individual is known with 100% certainty, whereas clinical group membership, for instance, is not. These, and other factors, including the complex mapping between brain and behavior whereby distinct neurobiological presentations can underlie similar behaviors and vice versa (Seghier & Price, 2018), make the challenge even more daunting when extending this and related approaches to studies of individual differences. Future studies will be needed to address these issues.

It is important to make it clear that we do not know to what extent, if at all, the specific connectome edges we identified, as maximally individually distinct might also be associated with individual differences in behavior. Further study of the minimal connectome subsets associated with behavioral variability rather than individual identity could reveal that the specific edges that maximally distinguish a particular behavioral measure may not be the same as those edges identified here as individually distinct. For instance, Noble et al. (2017b) have shown that the reliability of an edge (which is related to individual distinctness) is not linked with its usefulness in predicting one particular behavioral measure (IQ). More generally, the goals of predicting individual identity and predicting behavioral measures or groups can sometimes be at odds. The edges most effective at predicting group membership, for instance, will be maximally distinct between groups but must be similar within groups. There could thus be minimal overlap between the edges that distinguish groups and the edges that distinguish individuals, because the latter, by virtue of being individually distinct, may not be similar within group members. A characterization of the relationship between individually distinct and behaviorally distinct connectome edges—how many are needed for high-accuracy prediction, how diffusely they are distributed, and how many span different behavioral measures and how many are specific—will be important questions for future work.

We note that our general pattern of results is robust across our primary and replication datasets, numerous parcellations, temporal subsets of data, and across both resting-state and naturalistic viewing conditions. How minimal connectome subset sizes might change as a function of differing sample sizes and lower resolution data (Airan et al., 2016; Waller et al., 2017; Horien et al., 2018) is a question for future work. Furthermore, although a full exploration of thin-slice fingerprinting across different task conditions is beyond the current scope, a comparison between two different task conditions (rest and video-watching) in the replication dataset (Supporting Information Figure S4, Byrge & Kennedy, 2019) indicates that similarly high identification accuracy can be obtained in two task contexts by using similarly small numbers of edges, and that the individually diagnostic values of edges computed separately for each task context were broadly similar but not identical (Supporting Information Figure S9, Byrge & Kennedy, 2019). This is consistent with other work indicating similar but not the same patterns of functional connectivity across task contexts (e.g., Cole et al., 2014), and work showing largely similar numbers of principal components achieve optimal accuracy across different task conditions (Amico & Goñi, 2018; see also Finn et al., 2017, for a discussion of some of these issues).

In sum, we find that not only is a complete functional connectome not required for individual identification (see also Finn et al., 2015; Peña-Gómez et al., 2017; Amico & Goñi, 2018), but also that a remarkably small “thin slice” of the connectome is sufficient to identify individuals. Predicting identity on the basis of a targeted selection of the most individually distinct functional connections permits identification with the smallest subset possible (under 0.3% of connectome and fewer than 100 edges). However, many different connectome subsets permit high identification accuracy—including quite small randomly selected subsets (under 1% of connectome)—in part because individually distinct information is widespread across the cortex. Individualized brain signatures in high-dimensional fMRI datasets can thus be detected in a much more compact manner than previously appreciated, with potential implications for the underlying sources of that uniqueness as well. These results may also have implications for how one might approach developing brain-based measures that link to individual differences (see also Dubois & Adolphs, 2016), including the construction of biomarkers for psychiatric conditions.

## METHODS

### Participants

We primarily analyzed resting-state fMRI scans from the “S900 subjects release” of the Human Connectome Project (http://www.humanconnectome.org). Participant recruitment and consent is described in (Van Essen et al., 2013); briefly, informed consent was obtained from each participant, and the study protocol was approved by the institutional review board at Washington University in St. Louis, MO. We excluded participants with less than three resting-state scans available, leaving a final sample of *N* = 835 and 3,333 scans (828 participants with 4 scans; 7 with 3 scans). This dataset includes participants who are relatives; we also repeated the primary analysis using a randomly selected subsample of 282 unrelated individuals (1,125 scans; 279 subjects with 4 scans and 3 subjects with 3 scans).

As a replication dataset, we also examined a dataset collected at Indiana University and described in Byrge and Kennedy (2018) in which 54 adults (25 diagnosed with autism spectrum disorder [ASD]; 29 controls) participated. Informed consent was obtained from all participants; the local institutional review board at Indiana University approved the study protocol. This dataset consisted of resting-state scans and video-watching (i.e., naturalistic viewing) scans; we analyzed these scan types separately, treating them as different subdatasets. In the video-watching scans, participants watched sequences of movie trailers, which were different for each scan. After excluding participants with fewer than three rest or three video scans, the final sample consisted of scans from 48 participants (19 ASD), with 251 scans in the rest replication dataset (30 participants with 6 scans; 1 with 5; 15 with 4; 3 with 2) and 183 scans in the video replication dataset (42 participants with 4 scans; 3 with 3). No individuals in this sample were relatives.

### Data Acquisition and Preprocessing

MRI data acquisition and preprocessing is detailed in Smith et al. (2013) and Glasser et al. (2013) and briefly summarized here. Subjects participated in four resting-state scans across two sessions (2 scans per session, with opposite phase encoding directions, each approximately 14 minutes long [1,200 TRs]). Participants were instructed to remain awake with eyes open. MRI images were acquired using a customized 3 Tesla Siemens Skyra with 32-channel head coil. Parameters for T_2_*-weighted resting-state scans were as follows: TR/TE = 720/33.1 ms; 1,200 volumes; flip angle = 52°; 2-mm isotropic voxels; 72 slices; multiband acceleration factor of 8. High-resolution T_1_-weighted images of the whole brain were also acquired (MPRAGE, .7-mm isotropic voxel size; TR/TE/TI = 2,400/2.14/1,000 ms) as anatomical references.

We analyzed the “Resting State fMRI FIX-Denoised” release with preprocessing detailed in Smith et al. (2013) and Glasser et al. (2013); weak high-pass temporal filtering (>2,000 s FWHM) to remove slow drifts was performed. We regressed out the mean cortical signal (and its derivative) from the FIX-denoised BOLD data in a second step (Burgess et al., 2016; Byrge & Kennedy, 2018), and the residuals were analyzed as the cleaned data. Framewise displacement traces based on the movement parameters distributed with the data were computed via in-house MATLAB scripts following Power et al. (2012). We finally extracted BOLD time courses from the cleaned data as the mean signal across individual ROIs taken from numerous cortical parcellations.

Data acquisition and preprocessing for the replication dataset is described in Byrge and Kennedy (2018) and was designed to be similar to the HCP dataset. Briefly, four to six ∼16-min multiband resting-state scans per subject (TR = 813 ms; 1,200 TRs) and four video-watching scans (TR = 813 ms; 1,000, 952, 1,026, and 977 TRs) were acquired across two to three different days. Data was preprocessed using ICA-FIX followed by mean cortical signal regression, as described above. Weak high-pass temporal filtering was used for linear detrending.

### Cortical Parcellations

The primary parcellation examined was the 360-ROI HCP_MMP1.0 parcellation introduced by Glasser et al. (2016). We also examined symmetrical random parcellations of the cortical surface across numerous granularities (50, 100, 150, 200, 250, 300, 360, 400, and 500 ROIs). To create each random parcellation with granularity *g*, we used *ft_read_cifti* to import the HCP_MMP1.0 parcellation into MATLAB 2014b and then submitted the cortical surface coordinates or vertices (.pos data structure of imported cifti file) for the left hemisphere to the *k*-means algorithm (*kmeans* command in MATLAB 2014b), using a squared Euclidean distance measure, to obtain solutions with *k* = 1/2 *g* clusters, which we then mirrored to the right hemisphere. We created 100 random parcellations for each granularity by using this method.

We also estimated higher level functional network membership for each ROI by using the Yeo 7-network parcellation (Yeo et al., 2011). In brief, we defined the network assignment for a given ROI as the mode of the Yeo network assignments for each of the grayordinates comprising that ROI. More concretely, we used a mapping of resting-state networks to the cifti file format (RSN-networks.32k_fs_LR.dlabel.nii from https://balsa.wustl.edu/study/show/WG33) and used the HCP wb_command utility (*wb_command -cifti-all-labels-to-rois*) to convert the Yeo 7 network parcellation to a format that could be compared with the distributed HCP_MMP1.0 parcellation. The result was two assignments for each grayordinate, one assignment to one of the 360 HCP_MMP1.0 parcellation ROIs, and another to one of the Yeo 7 networks. Next, for each of the HCP_MMP1.0 parcellation ROIs, we extracted the Yeo 7 network assignment for each of the grayordinates assigned to that ROI and computed the mode, that is, the most frequently occurring Yeo 7 network across all the grayordinates in the given HCP_MMP1.0 ROI. This modal network was taken as the Yeo 7 network for the given HCP_MMP1.0 ROI. This mapping is available at brainlab.psych.indiana.edu/resources/hcp_mmp10_yeo7_modes.pdf for the interested reader.

For the replication dataset, the only parcellation examined was a 114-ROI cortical parcellation anatomically subdividing the Yeo 17 functional networks (Yeo et al., 2011) and described more fully in Betzel et al. (2014). For some visualizations, we assigned each of these ROIs to their best corresponding Yeo 7 functional network by using the following mapping from Yeo 17 network numbers to Yeo 7 network names (numbers): 1–2 to Visual (1); 3–4 to Somatomotor (2); 5–6 to Dorsal Attention (3); 7–8 to Ventral Attention (4); 9–10 to Limbic (5); 11–14 to Control (6); 15–17 to Default (7).

### Functional Connectome Construction

For each scan, we created functional connectivity (FC) matrices (“functional connectomes”) for each of the 901 parcellations examined.

Motion censoring/scrubbing prior to connectome construction was performed in all analyses. For all analyses unless stated otherwise, all TRs with FD >= 0.39 mm were censored, and all the remaining (uncensored) TRs were used to construct the connectivity matrices. Note that this means that the number of TRs used for constructing connectivity matrices varied across scans. We also conducted two Supporting Information analyses (Byrge & Kennedy, 2019) in which we altered the censoring procedure. In one, we constructed connectivity matrices after using a stricter censoring threshold (FD >= 0.2 mm) to examine whether the quality of included TRs might be influencing the pattern of results. In the other, we equated the number of TRs across scans to examine whether the quantity of included frames might be influencing the pattern of results. To do this, we excluded scans in which more than 30% of TRs (360) were censored (106 scans), and then for each remaining scan we randomly selected 840 “good” (uncensored) TRs to be used for connectome construction, ensuring that the same number of TRs was included for all scans. We repeated this random selection of 840 TRs 100 times. These alternate censoring procedures did not influence the pattern of results.

In all cases, using only the remaining TRs, we generated functional connectivity matrices by computing the Fisher *z*-transformed pairwise correlations among all ROI time series in the given parcellation. In contrast to other approaches (Finn et al., 2015, 2017), we analyzed one FC matrix for each scan, for a total of four matrices per subject, rather than averaging FC matrices acquired on the same day.

For the HCP_MMP1.0 parcellation only, we also examined FC matrices based on various fractions of the duration of the entire scan. We constructed FC matrices in the same manner as described above, except we did not include the entire length of the scan (1,200 TRs). Instead, we constructed FC matrices from contiguous fragments of the scan with the following durations, each increasing by approximately 1 min: 83, 167, 250, 333, 417, 500, 583, 667, 750, 833, 917, 1,000, and 1,083 TRs. For each such duration, we randomly varied the starting location 100 times or as many times as possible (18 times for 1,083 TRs; 100 times for all other durations), resulting in 1,218 temporally limited FC matrices for each scan.

Symmetric *g* × *g* FC matrices, for each parcellation granularity *g* (i.e., the number of ROIs) were reduced to 1/2 * *g* * (*g* − 1) × 1 FC vectors for use in subsequent analyses.

### General “Connectome Fingerprinting” Algorithm

We predicted individual subject identity from functional connectomes by using an approach based on the method originally introduced by Finn et al. (2015; 2017) and adapted for comparing four scans in one pass (vs. 2 scans as in Finn et al., 2015, 2017). In brief, we first computed the similarity between all pairs of scans as the correlation between the corresponding pair of FC vectors. Next, we predicted subject identity for a given scan by (a) identifying the scan with maximal similarity to the given scan, and (b) taking the subject identity corresponding to the maximally similar scan as the predicted subject identity for the given scan. Accuracy was computed as the percentage of scans for which the predicted subject identity was equal to the actual subject identity. We also conducted an even more stringent version of this same procedure in which we excluded scans from the same subject that were acquired on the same day (given that same-day scans would be expected to be more similar than different-day scans for a given subject; Birn et al., 2013), while retaining all available scans from other subjects (both same-day and different-day acquisitions). This across-day accuracy metric is presented in the Supporting Information (Byrge & Kennedy, 2019); highly similar results were obtained.

Accurate identification requires higher similarity of FC within individuals than across individuals; following Amico and Goñi (2018) we also separately examined within-individual similarity (i.e., the mean correlation among FC vectors from the same subject, or *I*_*self*_) and across-individual similarity (i.e., the mean correlation between FC vectors from different subjects, or *I*_*other*_).

In all the HCP dataset analyses presented, we employed a split-half design in which the 883 available subjects were randomly divided into a training partition and a test partition that were held fixed for all analyses. Exclusions based on data quality and available scans occurred after dividing the dataset, resulting in a training dataset with data from 421 subjects (418 with 4 scans and 3 with 3 scans for a total of 1,681 scans) and a test dataset with data from 414 subjects (410 with 4 scans and 4 with 3 scans for a total of 1,652 scans). Data from the 421 training partition subjects were used to rank the FC edges on the basis of individually diagnostic information (described below) but never used in the fingerprinting procedure itself. Data from the 414 test partition subjects were used in the fingerprinting procedure, such that for each scan, there were three possible true positive subject identifications (e.g., 3 other scans from the same subject, or 2 scans using the across-day metric) and up to 1,649 possible false positive subject identifications. Because some of the 883 available subjects were relatives and might have more similar functional connectomes (Miranda-Dominguez et al., 2017), we also repeated the primary analysis by using a randomly selected subset of 282 unrelated subjects, with 143 of the original training subjects (572 scans; 4 scans per subject) retained in the unrelated training partition and 139 of the original test subjects (553 scans; 136 subjects with 4 scans; 3 subjects with 3 scans) and retained in the unrelated test partition; results were unchanged.

For the replication dataset, a split-half design was also used, and the training partition and test partition contained equivalent proportions of ASD participants. The same participants were assigned to the same partitions in the rest and video analyses.

The fingerprinting procedure was always carried out separately within each parcellation or temporal duration or dataset, never comparing similarity of FC vectors across different parcellations or durations or datasets.

### Computing Individually Diagnostic Information (or Distinctness) for Each Functional Connection

We independently ranked functional connections (edges of the vectorized FC matrices) according to individually diagnostic information (or distinctness). Edges that are more diagnostic of individuals should have two properties: low within-subject variability and high across-subject variability. Thus, using FC vectors for the 414 training partition subjects only, we used a ratio of across-subject and within-subject variability to capture individually diagnostic value for each edge. We computed across-subject variability as the mean (across 4 scan sessions) of the standard deviation of the FC value for that edge across subjects within a given session, and within-subject variability as the mean (across subjects) of the standard deviation of the FC value for that edge across all sessions within each subject. We then ranked each edge according to this variability ratio from most to least individually diagnostic.

We note that the existing literature (Finn et al., 2015; Vanderwal et al., 2017) employs a different “differential power” ranking approach designed for comparing pairs of scan sessions; our approach is simpler and ranks multiple scans in one pass. The correlations between the differential power metric for different scan pairs and our variability ratio metric range from *r* = .63 to *r* = .66 for the HCP_MMP1.0 parcellation. Another alternative metric for evaluating the individual distinctness of FC edges is intraclass correlation (Shrout & Fleiss, 1979; Noble et al., 2017a, 2017b; Amico & Goñi, 2018), a measurement highly correlated with ours (*r* = 0.96, *p* << 0.001).

Individually diagnostic values were independently computed for each analysis conducted using parallel data (e.g., using the same parcellation, or the same temporal duration).

### “Thin-Slice” Connectome Fingerprinting Algorithm

To examine how much of the available information in the connectome is needed to accurately identify individuals, we conducted the general fingerprinting algorithm using various restricted subsets, or “thin slices,” of the vectorized FC matrix.

First, we attempted to identify individuals by using a subset of the connectome consisting of only the most individually distinct FC edges (as ranked by an independent partition of the data; see above). As a concrete example, we masked 99% of each vectorized FC matrix so that only the most individually diagnostic 1% of edges remained, producing a most distinct FC subset. Then, using these FC subsets from the test subjects only, we conducted the fingerprinting algorithm, predicting which individual contributed a given FC subset, based on maximal similarity with all other such FC subsets. We explored a complete range of percentages of the connectome, ranging from the top 0.01% of edges (where nearly the entire FC matrix is masked/not considered) to 95% (where only the least distinct 5% of edges is masked), sampling the percentage range more densely where needed to ascertain inflection points. This procedure is cumulative, such that when we predict identity by using the most distinct 2% of edges, the most distinct 1% of edges is also included; thus, both the number of edges included and the individually diagnostic value of edges included changes as the percentage is varied. We conducted this “thin-slice” fingerprinting procedure for each parcellation and temporal duration.

Next, we reversed this procedure, predicting individual identity by using connectome subsets that *omit* the most distinct edges. For instance, we predicted individual identity without using the most distinct 1% of edges, using instead a subset composed of the least distinct 99% of edges (see also Vanderwal et al., 2017, for a similar approach). We examined the same range of percentages as before, excluding the same percentage of the connectome that was included in the previous analysis.

In both of the preceding analyses, the size of the connectome subset used for prediction and the individually diagnostic value of the edges included in that subset covary. To decouple these factors, next, for the HCP_MMP1.0 parcellation only, we conducted a variant of this same procedure in which we kept the number of edges constant in each use of the algorithm while still varying the ranking of edges included from most to least distinct. To do this, we repeated the “thin-slice” fingerprinting algorithm by using one quantile of the independently ranked edges at a time. In contrast to the previous analyses, this procedure is not cumulative, such that the second quantile does not include edges that fall within the first quantile. We explored a range of *q*-quantile sizes corresponding to 0.02% of the connectome (subsets of 13 edges; *q* = 5,000) to 1% of the connectome (subsets of 646 edges; *q* = 100) and then scaled all quantiles to a maximum of 100 for comparability (although, note that most quantiles examined were smaller than percentiles).

We next examined “thin-slice” fingerprinting accuracy when the connectome subset contains randomly-selected edges, ignoring the ranking of individually diagnostic value. Here, for each of the percentages explored in the first “thin-slice” analysis, we randomly selected the same number of edges without considering individually diagnostic value, and obtained fingerprinting accuracy by using the connectome subset corresponding to those edges. We conducted 100 random selections for each connectome percentage examined.

Finally, we predicted individual identity by using connectome subsets defined at the network level using the mapping between the 360 HCP_MMP1.0 parcellation ROIs and seven functional networks (Yeo et al., 2011) described previously. For each network, we predicted individual identity by using subsets composed of only within-network edges as well as using only within- and across-network edges. Then, taking a virtual lesion approach, for each network, we predicted identity by using the entire connectome *except* within-network edges for that network, as well as using the entire connectome except within- and across-network edges for that network.

## ACKNOWLEDGMENTS

This work was supported by the NIH (R01MH110630 and R00MH094409 to DPK), and NICHD (T32HD007475 Postdoctoral Traineeship to LB). For supercomputing resources, this work was supported in part by Lilly Endowment, Inc., through its support for the Indiana University Pervasive Technology Institute, and in part by the Indiana METACyt Initiative. The Indiana METACyt Initiative at IU was also supported in part by Lilly Endowment, Inc. We thank the Human Connectome Project for making their data available. Data were provided in part by the Human Connectome Project, WU-Minn Consortium (Principal Investigators: David Van Essen and Kamil Ugurbil; 1U54MH091657) funded by the 16 NIH Institutes and Centers that support the NIH Blueprint for Neuroscience Research; and by the McDonnell Center for Systems Neuroscience at Washington University. For the replication dataset collection and analysis, we thank Hu Cheng for MRI protocol development, Soyoung Park for training the FIX classifier, and Brad Caron and Susannah Burkholder for data collection. We also thank Olaf Sporns for helpful discussions.

## AUTHOR CONTRIBUTIONS

Lisa Byrge: Conceptualization; Formal analysis; Visualization; Writing – original draft; Writing – review & editing. Daniel P. Kennedy: Conceptualization; Funding acquisition; Supervision; Writing – review & editing.

## FUNDING INFORMATION

Daniel P. Kennedy, National Institutes of Health (http://dx.doi.org/10.13039/100000002), Award ID: R01MH110630. Daniel P. Kennedy, National Institutes of Health (http://dx.doi.org/10.13039/100000002), Award ID: R00MH094409. Linda B. Smith, National Institute of Child Health and Human Development (http://dx.doi.org/10.13039/100000071), Award ID: T32HD007475, postdoctoral traineeship to Lisa Byrge.

## TECHNICAL TERMS

Functional connectome: The set of all functional connections associated with a given fMRI scan and a given parcellation.
Functional connection (edge): The extent to which the measured activity of two different brain regions is coordinated, as assessed via correlation.
Fingerprinting algorithm: Predicting which participant contributed an fMRI scan based on relative similarity across functional connectomes.
Functional network: Distributed brain regions whose activity is coordinated.
Thin-slicing: Making inferences from minimal amounts of information (often from brief experiences) that can be highly accurate.
Parcellation: Subdivision of the brain (typically based on neuroanatomy or function) into regions of interest within which brain activity is measured.
HCP_MMP1.0 parcellation: State-of-the-art neuroanatomically and neurofunctionally informed group-level 360-ROI parcellation of the cortex.
