# Supplementary material for: High-accuracy individual identification using a “thin slice” of the functional connectome
Source: Netw Neurosci. 2019 Feb 1;3(2):363–83. doi: 10.1162/netn_a_00068 (PMC6370471; doi:10.1162/netn_a_00068)
Supplement: Supplementary file 1 [file netn-03-363-s001.pdf]

Byrge, L. & Kennedy, D. P. (2019). Supporting Information for “High-accuracy individual identification using a “thin slice” of the functional connectome.” *Network Neuroscience*, *3*(2), 363–383 . https://doi.org/10.1162/netn_a_00068

Supplemental Information

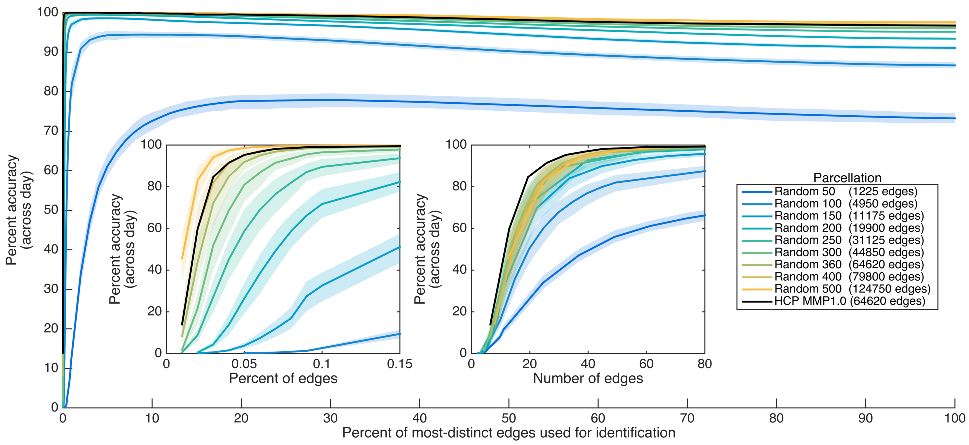

Supplemental Figure 1. *Identification accuracy as a function of how many of the most individually distinct edges in the functional connectome are used for identification, across different parcellations, expressed in terms of the percentage of the complete connectome included in each subset. This plot corresponds to Figure 1a in the main text but uses a more stringent accuracy metric, in which only scans from the same individual that were acquired on a different day are counted as a success. Despite generally lower accuracy as expected, the pattern of results is effectively the same. 99% confidence intervals based on a bootstrap estimate of the mean across five random parcellations of the same resolution are depicted for each random parcellation resolution. Insets depict the main figure rescaled to reveal very small x axis values.*

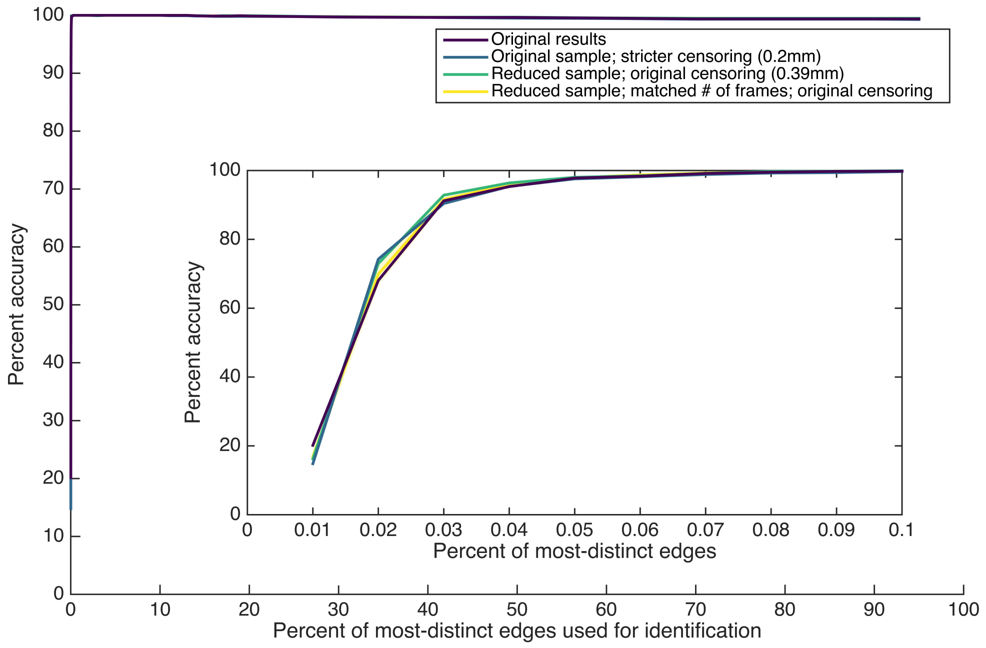

Supplemental Figure 2. *Identification accuracy as a function of how many of the most individually distinct edges in the connectome are used for identification, using the HCP_MMP1.0 parcellation only, examining the results of different data quality thresholds to ensure that head motion is not driving the effect. The purple line is repeated from Fig. 1a for comparison purposes. It includes the complete original sample of participants, in which frames exceeding 0.39mm were scrubbed/censored prior to connectome construction. The blue line plots the results obtained when re-running this original analysis using a stricter censoring threshold (0.2mm) and the same sample. Note that the purple and blue lines are virtually the same, indicating that the results are not driven by the quality of frames included in the analysis. To ensure that the number of frames included, which varied across scans in the preceding analyses, is not driving the pattern of results, we repeated the primary analysis using*

functional connectivity matrices constructed from randomly downsampled scans such that the same number of 'good' (uncensored) frames were included in each scan (840 TRs; 70% of the scan). This analysis was run 100 times with different randomly downsampled functional connectivity matrices; the yellow line plots the mean across all results along with 99% confidence intervals based on a bootstrap estimate of the mean. This analysis required a slightly reduced sample in which scans with fewer than 840 uncensored TRs were excluded (52 excluded from training partition; 54 excluded from test partition); we also re-ran the primary analysis using that same reduced subsample with no downsampling of frames (and the original censoring criterion, 0.39mm); this is plotted in green. Note that the yellow and green lines are effectively the same, indicating the number of frames included is not driving the pattern of results. The inset depicts the main plot rescaled to reveal small x axis values. In both the main and inset figures, four lines and confidence intervals for one line are plotted but largely overlap. The accuracy metric used for all these analyses was the original metric used in Figure 1 and throughout the main figures. For all new analyses presented here (yellow, green, and blue lines), the complete analysis was re-run, e.g. first the FC edges were re-ranked according to distinctness in the training partition with the relevant data quality thresholds applied, and then individual identity in the test partition was predicted using those edges.

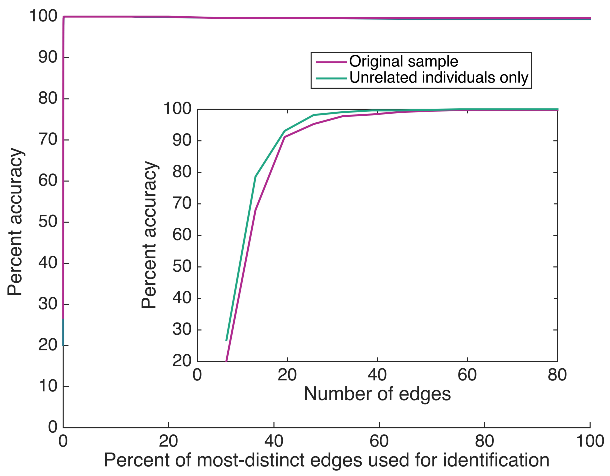

Supplemental Figure 3. *Identification accuracy as a function of how many of the most individually distinct edges in the connectome are used for identification, using the HCP_MMP1.0 parcellation only, conducted using a randomly selected subsample of 282 unrelated participants (green). Individually diagnostic values for each connectome edge were re-computed within this sample (using 143 of the original training partition participants) and those values were used to rank the edges in the identification procedure (using 139 of the original test partition participants). Identification performance for the original complete sample is re-plotted from Fig. 1a for comparison purposes (pink). The pattern of results is the same; family structure in the original sample is not driving the results.*

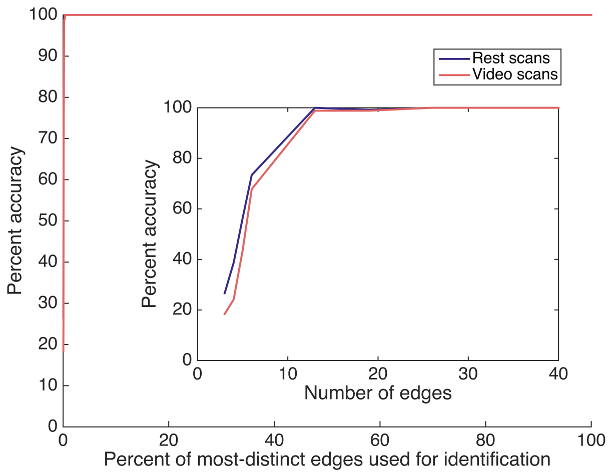

Supplemental Figure 4. *Identification accuracy in the replication dataset collected at Indiana University. Edge ranking and identity prediction were carried out separately among rest scans (purple line) and video-watching scans (orange line), using the same assignment of participants to the training partition or test partition. Accuracy is presented as a function of how many of the most distinct edges in the functional connectome used for identification and use a subparcellation (described in Betzel et al., 2014) of the Yeo 17 functional network parcellation (Yeo et al., 2011) into 114 cortical ROIs. Inset depicts the main figure rescaled to reveal very small x axis values. See Supplemental Figure 9 for more detail about the rankings of edges.*

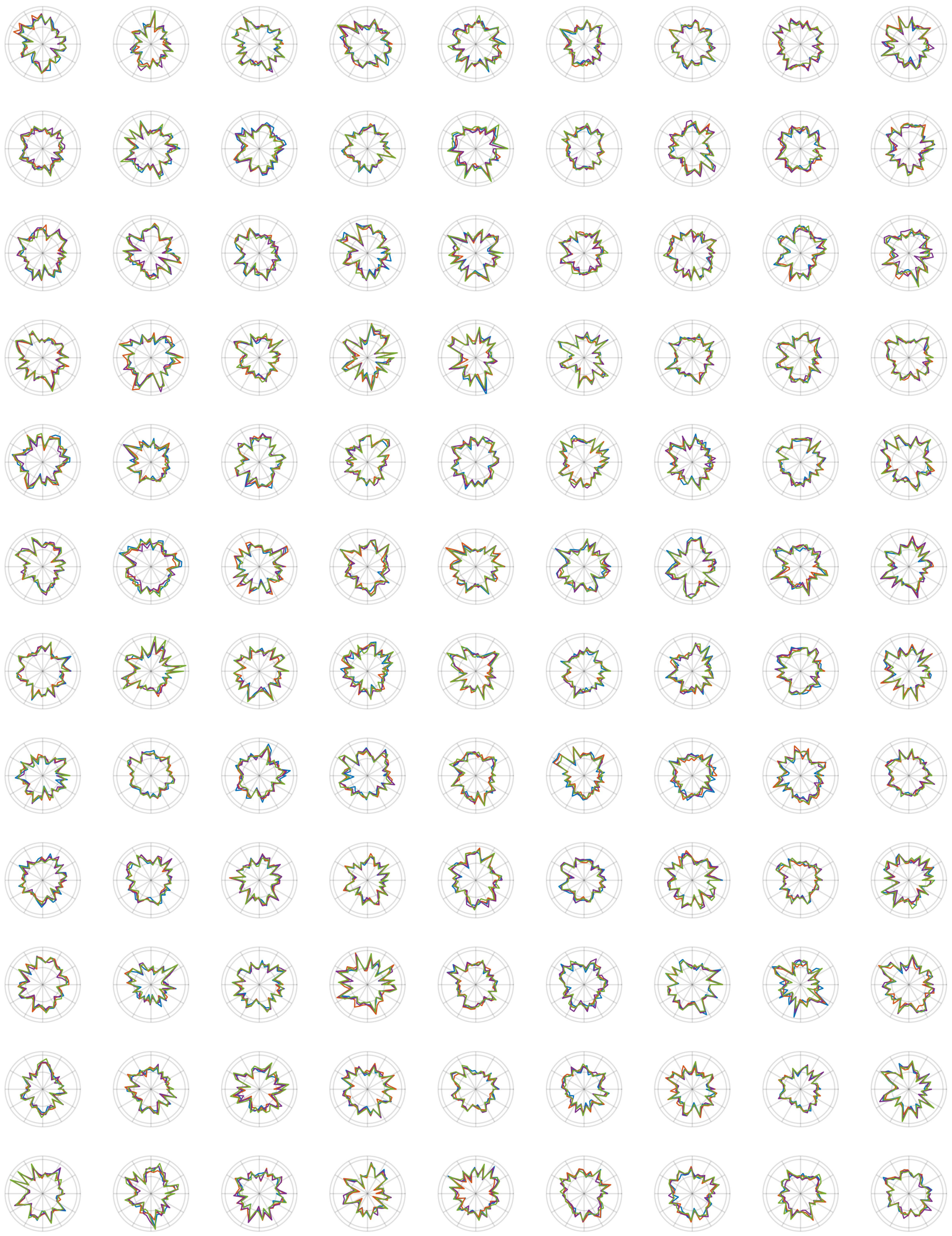

***Supplemental Figure 5**. Distinct patterns of functional connectivity within randomly-selected individual subjects. Each plot presents data from one subject, with data from different scans plotted in different colors. All plots depict functional connectivity values (Fisher z-transformed correlations) for the 40 edges with highest individual*

distinctness. The radius extends from -0.5 at center to a maximum of 1.2; grid lines indicate 0, 0.5, and 1. Please refer to Figure 2 for a legend.

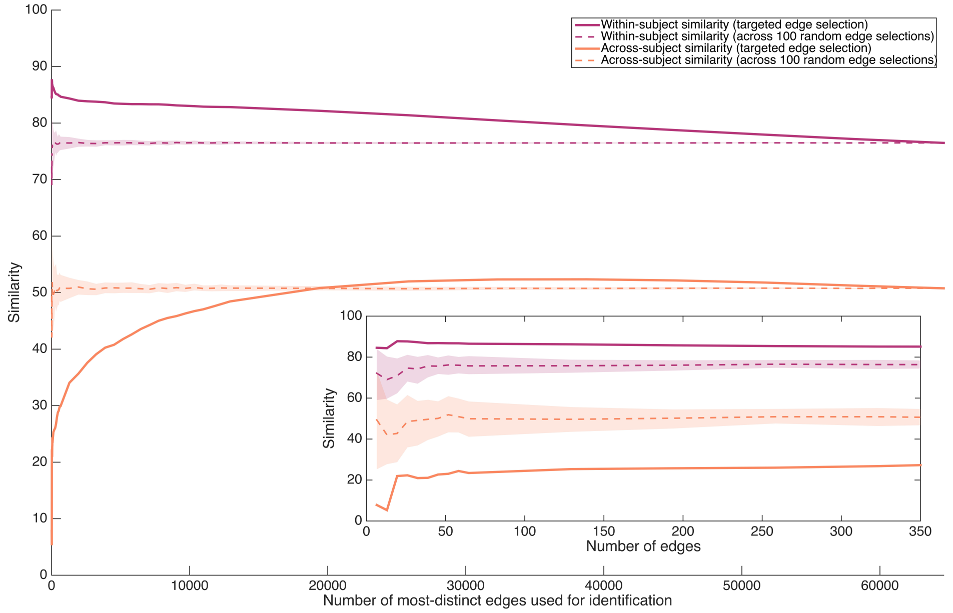

Supplemental Figure 6*. Mean within-subject similarity (self-similarity; maroon solid line) and across-subject similarity (other-similarity; orange solid line) as a function of how many of the most individually distinct edges in the connectome are used for identification, using the HCP_MMP1.0 parcellation only. The mean similarity values (correlations) are multiplied by 100 for comparability and correspond to* I_{self} *and* I_{other} *in Amico & Goñi (2018). Within-subject similarity is generally high for all connectome sizes and the gap between within- and across-subject similarity is evident. The dotted lines present the mean within- and across- subject similarity across random edge selections for each subset size examined (corresponding to Figure 5) along with 99% confidence intervals based on a bootstrap estimate of the mean across 5 (out of 100) random edge selections for each subset size examined. Note that targeted edge selections based on individually diagnostic values (solid lines) initially achieve, as*

designed, higher within-subject similarity and lower across-subject similarity, and then with increasing subset sizes approach the similarity values obtained for random edge selections (dotted lines). The inset presents the same plot re-scaled to reveal small x axis values.

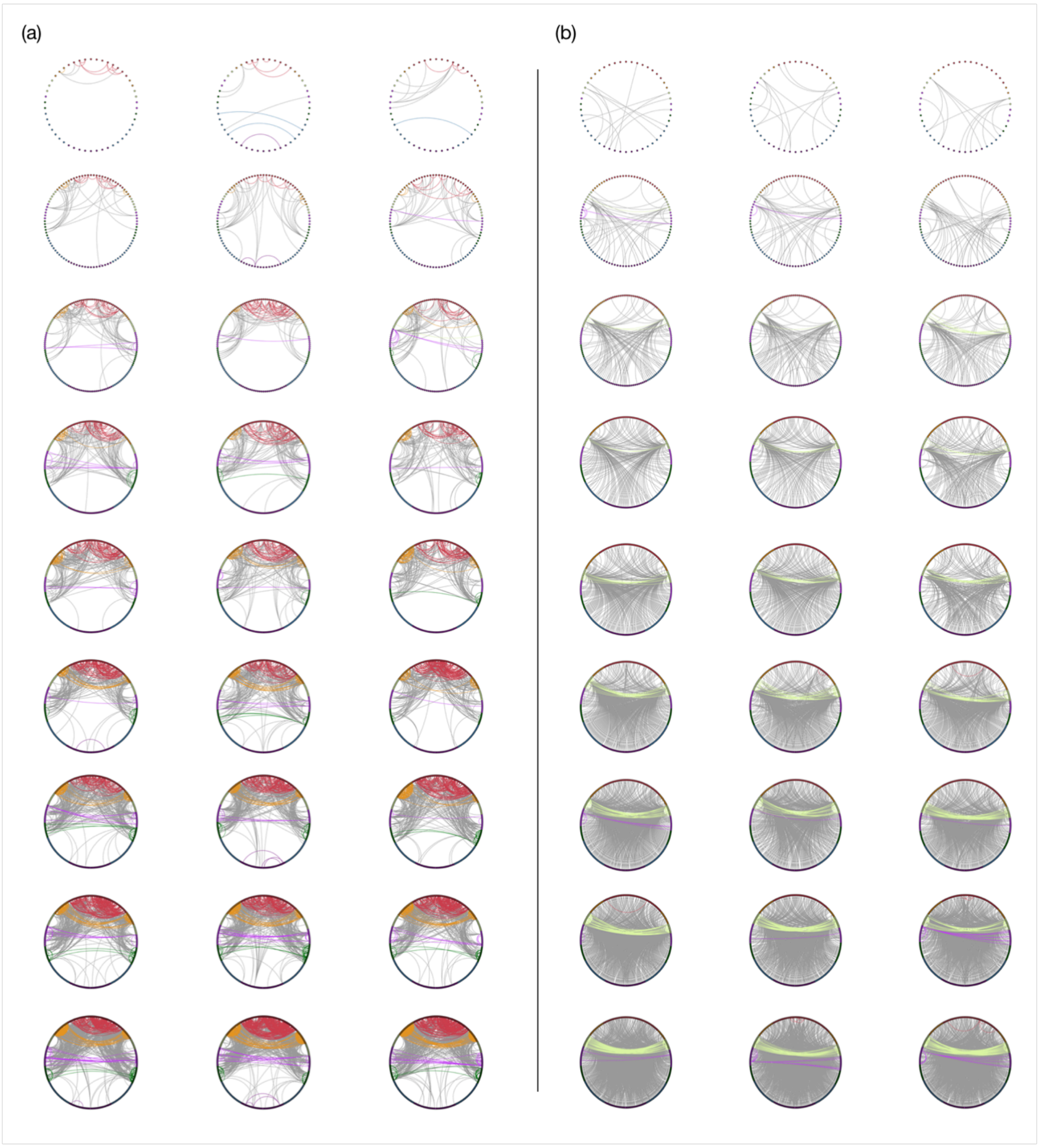

Supplemental Figure 7. *Circle plots visualizing the 1% of edges with the highest (left column) and lowest (right column) individually diagnostic value. Each row contains 3 random parcellations of the same size, selected at random, with parcellation granularity increasing from 50 (top row) to 500 (bottom row). Nodes and within-network edges are in color corresponding to large-scale functional network membership (refer to Figure 2 for color legend); across-network edges are in gray.*

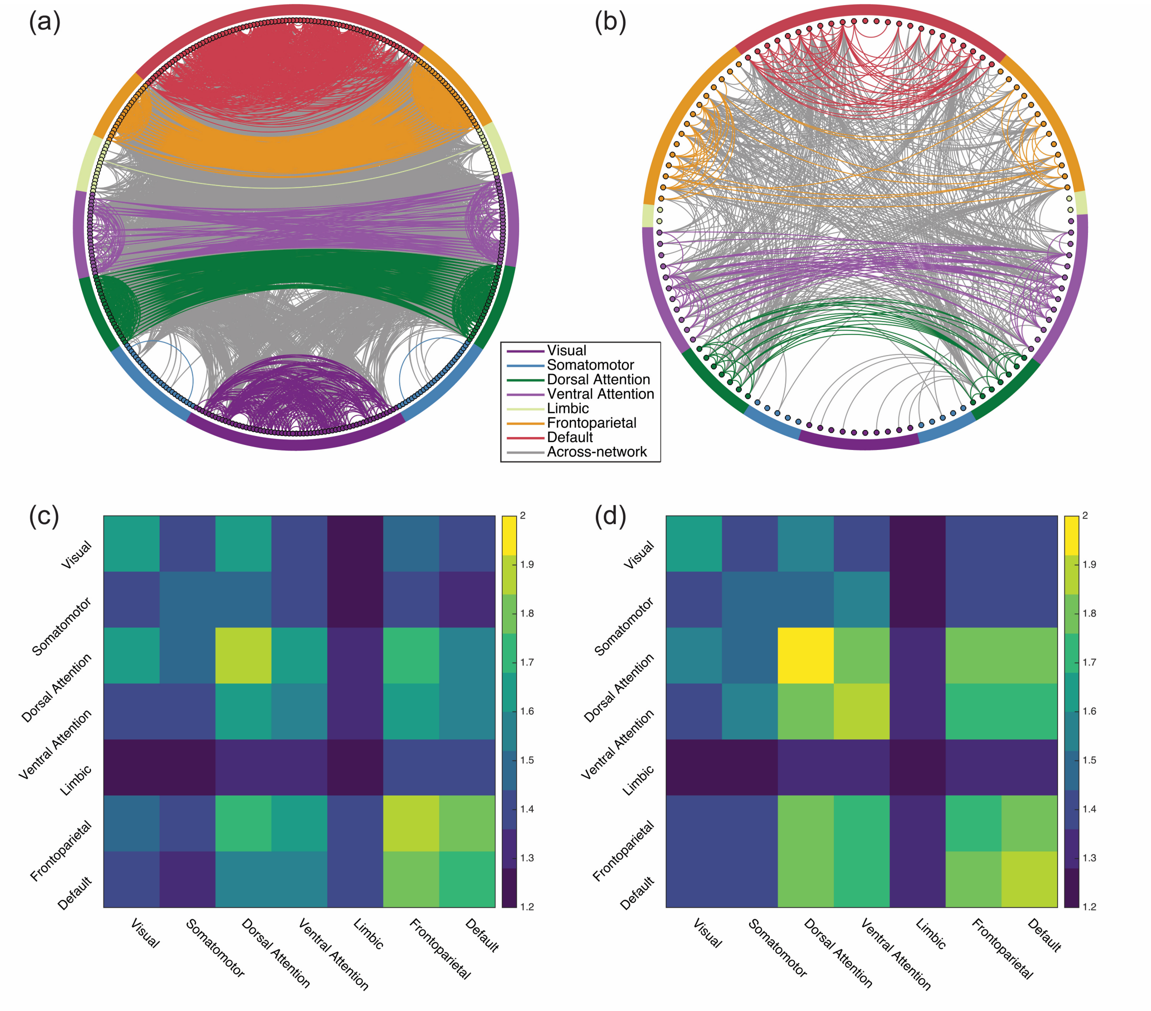

Supplemental Figure 8. *Individually diagnostic values for the primary dataset with HCP_MMP1.0 parcellation (left) and replication rest dataset (right). Top: circle plots visualizing the 10% of edges with the highest individually diagnostic value in each dataset, for visual comparison only, for HCP (a) and replication rest (b) datasets. Bottom: mean individually diagnostic value of all edges within each high-level functional network (diagonal) and between each pair of high-level functional networks (off-diagonal), for HCP (b) and replication rest dataset (d). These two matrices are highly correlated (r = 0.89, p* $<<$ 0.001*) suggesting broadly similar rankings at the high-level network level across these two datasets. However, note that*

edges cannot be compared directly across these two datasets due to format differences (volumetric vs. surface based), and that there are considerable differences in the parcellation granularities (360 ROIs vs. 114 ROIs) as well as the number of edges assigned to specific functional networks (c.f. Limbic network, represented by relatively more edges in the primary dataset than in the replication dataset).

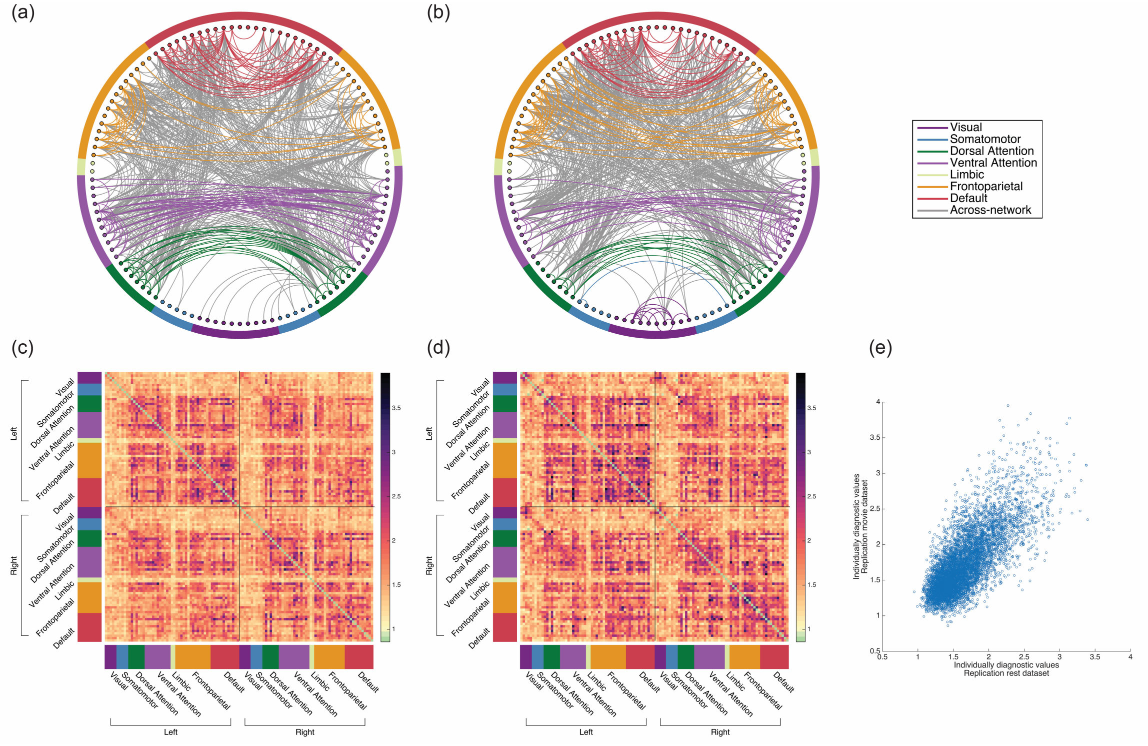

Supplemental Figure 9. *Individually diagnostic values in the replication dataset for the rest scans (left) and video-watching scans (center). Ranking of edges (and identity prediction) was conducted separately using only the rest scans and only the video-watching scans, using the same assignment of subjects to the training or test partition. Top: edges in the highest 10% of individually diagnostic value (a: rest; b: video). Considerable similarities are apparent, as well as differences (note that visual and somatomotor edges are among the top-ranked edges for the video-watching scans but not the rest scans). Bottom: individually diagnostic values for all edges (c: rest; d: video) which were similar across scan types (e; r = 0.74, p << 0.001). Note that (a) is repeated from Supp. Fig. 8b for comparison purposes.*

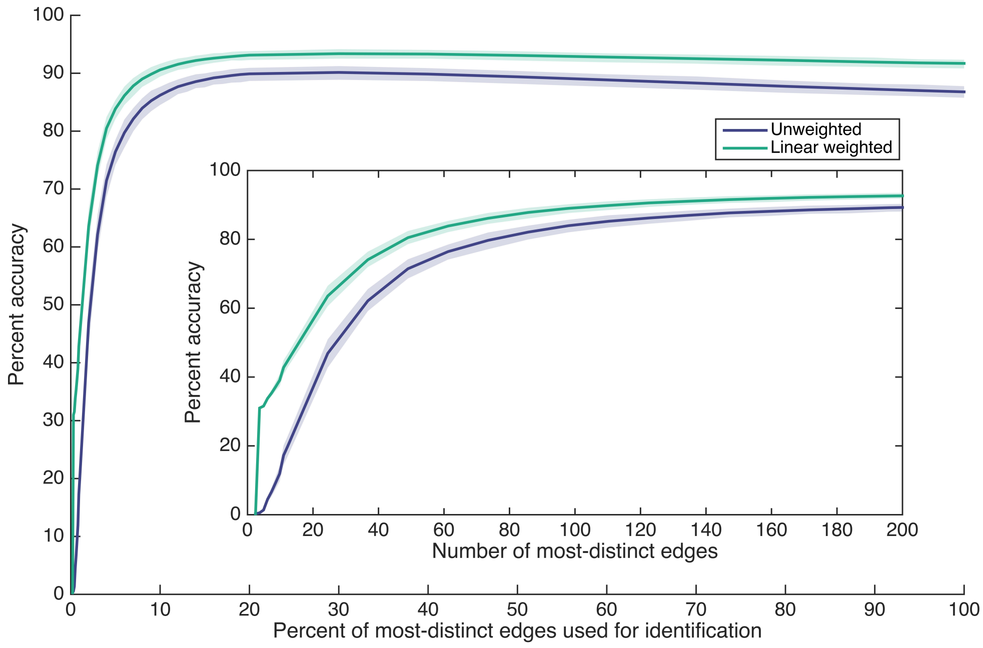

Supplemental Figure 10*. Identification accuracy using random parcellations of 50 ROIs, with identification conducted after linearly weighting edges according to their independently-determined individually diagnostic value (green). Individually diagnostic value was scaled from 0-1 prior to using as weights. As before, accuracy is presented as a function of how many of the most distinct edges in the functional connectome are used for identification. Unweighted identification accuracy in this same parcellation, originally presented in Fig. 1a, is re-plotted here for comparison (blue). 99% confidence intervals based on a bootstrap estimate of the mean across five random parcellations are depicted for each line.*
